# The 988 suicide hotline—Lifeline or letdown? A pre-post policy analysis

**DOI:** 10.3389/fpubh.2024.1337362

**Published:** 2024-04-17

**Authors:** Michaella Baker, Juliet Sorensen

**Affiliations:** Pritzker School of Law, Northwestern University, Chicago, IL, United States

**Keywords:** suicide, 988, hotline, Lifeline, policy, mental health, legislation

## Abstract

Suicide has emerged as an urgent threat in recent years as COVID-19 impaired the health and economic wellbeing of millions of Americans. According to the Centers for Disease Control and Prevention, the impact of COVID-19 and the ongoing opioid epidemic has “taken a mental, emotional, physical, and economic toll on individuals, families, and communities,” increasing the need for innovative solutions to prevent suicide on a national scale. The National Suicide Hotline Designation Act of 2020 established 988 as the universal telephone number for suicide prevention and represents a key federal intervention to address this crisis. However, research on 9-8-8's effectiveness is limited, given the Act's recent enactment and implementation at the federal and state levels. This policy analysis investigates how and to what extent the mental health crisis system in Georgia has improved since the implementation of the 2020 Act as well as the implications of state law on population-level mental health outcomes. Georgia is used as a nationally representative case study for two reasons: (1) Georgia had a robust statewide suicide hotline prior to 2020, providing solid infrastructure on which federal expansion of a suicide hotline number could be built, and (2) the conflicting characteristics of Georgia's mental health system represent several different pockets of the U.S., allowing this analysis to apply to a broad range of states and locales. The paper draws on takeaways from Georgia to propose state and national policy recommendations for equitable interventions to prevent and respond to this form of violence.

## Introduction

Suicide is an urgent threat to public health and the lives of millions of Americans (1). While suicide has presented a significant challenge in the United States for years, suicide rates have increased dramatically in the past two decades. From 2000 to 2020, suicide rates rose by 30% (2). In 2021 alone 1 year into the COVID-19 pandemic-−12.3 million American adults age 18 and older seriously thought about suicide, 3.5 million planned suicide, and 1.7 million attempted suicide (3). According to the Centers for Disease Control and Prevention (CDC), the impact of COVID-19 and the ongoing opioid epidemic has “taken a mental, emotional, physical, and economic toll on individuals, families, and communities,” increasing the need for innovative solutions to prevent suicide on a national scale (4).

However, suicide only represents a portion of the problem. The U.S. lacks a robust mental health crisis system that can provide immediate de-escalation services to assist someone experiencing a mental health crisis. Americans are substantially more likely to receive treatment in ambulatory settings like emergency departments (EDs) for non-fatal self-harm, like suicide attempts, than in mental health treatment centers and hospitals (4). From 2008 to 2017, the rate of ED visits related to suicidal ideation or suicide attempts rose consistently for all age groups (5). Unfortunately, EDs are often not equipped to address mental health crises, given the often over-crowded, over-stimulating, and time-pressured environment and limited qualified mental health staff. As a result, people often wait hours or even days to access care when they may not have the luxury of time (6).

Before individuals in mental health crises arrive in an ED, they often interact with law enforcement, mainly because their caregivers or bystanders have few options in times of crisis other than calling 911. As a result, roughly one in 10 individuals with mental health disorders have interacted with law enforcement before receiving any kind of psychiatric care (7). The interaction with law enforcement almost always takes place in a police vehicle, many times in handcuffs—a scenario that contradicts the central tenets (safety, trustworthiness, choice, collaboration, and empowerment) (8) of trauma-informed mental health care (9). Police involvement has been found to escalate the presenting situation, intensify distress, and increase public stigma and criminalization of mental illness.

The National Suicide Hotline Designation Act of 2020 aimed to change this cascade of events. In October 2020, the Federal Communications Commission (FCC) and Congress designated a new three-digit dialing code (988) for Americans to reach the National Suicide Prevention Lifeline (Lifeline) and required states to adopt the Lifeline by July 16, 2022 (10). 988 was intended to build on the infrastructure of the existing toll-free Lifeline number (1-800-273-TALK) but with an easier-to-remember number and broader directive: to provide 24/7 phone or text support for anyone experiencing a mental health crisis or in need of suicide prevention services (9). Thus, rather than calling 911 when someone is in mental distress, 988 enables access to mental health crisis support without involving law enforcement. This not only has the potential to decriminalize mental health care, but also it empowers individuals to avoid unnecessary law enforcement and medical emergency department visits and to initiate psychiatric assessment and treatment sooner (9). In fact, early research in Tuscan, Arizona revealed that 80% of Lifeline calls were resolved without dispatching mobile crisis teams, law enforcement, or emergency medical services, showcasing the advantages of a robust crisis system (3).

In the 6 months after the launch of 988 on July 16, 2022, the Lifeline received over 2.1 million contacts—consisting of over 1.43 million calls, over 416,000 chats, and more than 281,000 texts (11). While the volume of calls and texts is encouraging, the potential for 988 to improve mental health care can only be effective if it operates in a linked fashion, not only providing a number to call, text, and chat but also services along a continuum of care. In other words, a trained call-taker must exist on the other end of the line to de-escalate a crisis and have access to mobile crisis teams with specialists trained for the job as well as mobile crisis centers. Without each step along the continuum of care, states cannot provide a reliable response for 988 callers.

While the federal government made investments in 988's launch and implementation, the responsibility for ongoing funding depends on state and local governments. However, to date, states have been slow to adopt legislation to address the increased demand in callers seeking mental health services. For example, the 2020 Act specified that states may collect cellphone fees on customers' phone bills, similar to 911, to sustainably fund the local 988 call centers. However, only eight states have enacted such legislation (California, Colorado, Nevada, Oregon, Virginia, Delaware, Washington, and Minnesota) (12, 13). Five states are approaching final legislative approval (Ohio, West Virginia, Maryland, Pennsylvania, and New York) (13). Trust fund appropriations have been set up by some states to support 988 crisis centers including Illinois, Indiana, Utah, and New Hampshire, but it is unclear whether these initiatives can support 988 and corresponding crisis centers' long-term funding needs (12, 13). The 2020 Act created a path forward for states to bolster their continuum of mental health crisis services, yet determining the accessibility, quality, and impact of 988 requires additional research into states' crisis systems.

### Research objectives

Given that the effectiveness of 988 will vary by state, this paper will focus on Georgia's legislative actions and policies pertaining to suicide prevention. Georgia provides an interesting case study for two key reasons. First, Georgia had a robust statewide suicide hotline prior to 2020, providing solid infrastructure on which federal expansion of a suicide hotline number could be built. In fact, the Substance Abuse and Mental Health Services Administration (SAMHSA) poached Monica Johnson, who served as the Interim Commissioner for the Georgia Department of Behavioral Health & Developmental Disabilities, to lead the 988 & Behavioral Health Crisis Coordinating Office because of her experience overseeing and successfully implementing programs funded through SAMHSA in Georgia (14). Evaluating Georgia will allow us to consider the impact of 988 in a system potentially more advanced and with more resources than other states. The successes and shortcomings Georgia has experienced in scaling up its Lifeline can serve as both a lesson and a warning for states starting from scratch.

Second, when it comes to the state's mental health response, Georgia is an early adopter in some respects and a late adopter in others. The conflicting characteristics of Georgia's mental health system represent several different pockets of the U.S., allowing this analysis to apply to a broad range of states and locales. For example, despite its current crisis response and suicide prevention infrastructure, in 2021, Georgia had one of the highest percentages of 988 call abandonment, which represents a shortfall in answering calls from help-seekers (15). Especially given that 988 is advertised on nearly every CDC and Georgia state health webpage that mentions mental health or substance abuse, it is essential to know whether the Lifeline's performance has improved since new legislation passed and the interplay of federal vs. state governments in scaling up state-level planning (16).

Further, Georgia's mental health coverage gap is significant. Georgia has an uninsured rate of 13.7%, which is the third highest in the country (17). If 988 calls are dropped, many Georgians would have nowhere to receive mental health services, as many do not have access to insurance that covers any health expenditure, let alone behavioral health. Because the state has opted not to accept federal funding to expand its Medicaid program under the Affordable Care Act (ACA), many people are too poor to obtain private health insurance but not poor enough to receive coverage under Medicaid. Closing the mental health coverage gap by providing affordable, accessible mental health care can significantly strengthen mental health and addiction treatment and services in the state.

In this paper, we begin with an overview of national suicide prevention efforts in the U.S. over the past 50 years and the reforms and legislation that made way for the National Suicide Hotline Act of 2020 and its subsequent implementation. We then detail how 988 operates on a national and state level to set the foundation for a Georgia-specific analysis. The pre-post policy analysis will evaluate Georgia's statewide suicide hotline number and the federal Lifeline before the National Suicide Hotline Designation Act of 2020 was passed and whether the performance of the Lifeline improved after the Act's passage and implementation. The paper ends with policy recommendations and a call to action—to be applied to Georgia and the U.S. broadly highlighting effective interventions to prevent and respond to this form of violence.

### A brief history of national suicide prevention efforts

The U.S. has historically neglected to implement a robust mental health crisis system. Divisions in responsibility between the states and federal government coordination problems rooted in federalism and separation of powers—exacerbate the lack of effective legislation and response (18). For example, President Kennedy's Community Mental Health Centers Act of 1963 (19). stipulated federal funding for community mental health centers to provide crisis care—yet most communities never received funds (20). Kennedy envisioned that each state would build mental health facilities based on geographic availability, state-specific inpatient and outpatient needs, and deployment of professional staff, with virtually no federal oversight (21). However, requirements for crisis care all but disappeared when the program was converted to a block grant in 1981 (18). Without a federal directive and funding, most states did not develop adaptive solutions. The Community Mental Health Centers Act illustrates the challenges created by federalism. A national structure that requires state-led implementation creates an inherent tension between policy and practice: states have a level of autonomy to direct policy initiatives within a national statutory scheme.

Nonetheless, in the early-80s, suicide prevention started to garner national attention. In 1983, the CDC established a violence prevention unit to spotlight rising youth suicide rates (22). A few years later, the HHS Secretary established a Task Force on Youth Suicide, which reviewed existing evidence and issued recommendations (22). However, it was not until the mid-1990s that suicide became a central issue, marked with two congressional resolutions—S. Res. 84 and H. Res. 212 of the 105^th^ Congress—which recognized suicide prevention as a national priority (22). Building on this momentum, in 1999, Surgeon General David Satcher issued *The Surgeon General's Call to Action to Prevent Suicide*, which introduced the blueprint for suicide prevention in the U.S. and called for a comprehensive national strategy (1), and in 2005, SAMHSA established the National Suicide Prevention Lifeline (1-800-273-TALK) (3).

SAMHSA aimed for 1-800-273-TALK to serve people at risk of suicide by providing de-escalation services through a national network of local call centers (3). This objective was based on early research showing that telephone crisis services reduced the crisis state of callers in mental distress (23). In the years after the Lifeline was established, researchers established proof of concept (24). Madelyn Gould, a psychiatric epidemiologist at Columbia University, assessed 1,085 callers between 2003 and 2004 expressing suicidality to the Lifeline call center and found a significant decrease in suicidality, psychological pain, and hopelessness during the telephone session (25). Benefits persisted when researchers interviewed a sample of the same callers within 3 weeks after the initial call (25). Nonetheless, nearly 10 years after SAMHSA instituted the Lifeline, analyses found it was underutilized, and the quality of services varied widely (26). While there were several national strategies and federal policy initiatives in the ensuing years (22), the rate of suicide across the U.S. continued to increase (27). The nation still had no template for what crisis systems should look like (1).

In 2016, the landscape changed. A Task Force of the National Action Alliance for Suicide Prevention (the “Task Force”) surveyed best practices across the country and found that to achieve optimal results, a more robust system of crisis care was needed on a state and regional basis (28). The Task Force recommended that this system include regional or statewide call centers, mobile crisis teams, and crisis care facilities. New research expanded the understanding of how to implement equitable suicide prevention practices in healthcare systems and communities. New partners became engaged in suicide prevention, including organizations and businesses not previously invested in suicide prevention, such as Instagram and its parent company, Facebook (29). Although funding still did not reflect suicide's grave and wide-reaching impact, throughout the late 2010s, suicide prevention efforts expanded and multiplied (1).

### Paving the way for the 2020 National Suicide Hotline Act

The aforementioned trends made way for the FCC to propose 988 as the three-digit telephone number for national suicide prevention and mental health crisis in 2019. The following year, the National Suicide Hotline Act (30) was signed into law, incorporating 988 as the new Lifeline. Building on the infrastructure established in 2005, the legislation provided an updated framework to run 988. For example, it required 988 to become operational in all states by July 16, 2022, via call, text, and chat. It instructed that states finance call centers using state, local, and private funding, and enabled states to add a fee to phone bills, much like 911, to expand, support, and improve 988 services (3). It also designated Vibrant Emotional Health (Vibrant) (31)—a non-profit organization that offers confidential emotional support through state-of-the-art crisis services and contact center operations to oversee the Lifeline through a cooperative agreement administered by SAMHSA (3). Like Kennedy's 1963 Community Mental Health Act, the Act was the equivalent of a national directive that requires state-led implementation.

Operationally, the FCC proposed that 988 would route callers experiencing a mental health crisis to the nearest local call center based on area code. If the local crisis call center is unavailable or the wait is too long, callers are redirected to a subnetwork of contracted national backup call centers. Federal funds were also used to augment text and chatting capabilities at national call centers, as local caller centers are not equipped to address this form of outreach (3). Still, despite the overflow system and specialized text and chat centers, as of 2020, Lifeline capacity was only sufficient to address approximately 85% of calls, 56% of texts, and 30% of chats (3).

HHS published an updated *Call to Action* in 2021, which further substantiated the case for 988. The report advocated for improving crisis infrastructure to enable 988 to triage calls, deliver important phone intervention services, and coordinate connections to additional support. Specifically, HHS called for the federal government to address “gaps, opportunities, and source needs to achieve standardization across crisis centers,” optimize “systems financing for 988 as the hub of an enhanced, coordinated crisis system,” and encourage “health care insurers to provide reimbursement for crisis services” (1).

Upon implementation in July 2022, 988 was connected to a network of over 200 local- and state-funded crisis centers (12). Although the Lifeline had been fielding calls through 1-800-273-TALK since 2005, call centers observed an immediate spike after 988 implementation. The combined number of calls, texts, and chats into 988 increased by 43%, with text volume alone representing a 700% increase compared to the year prior (12). As of December 2022, people who reached out to 988 spent less time waiting on hold for a counselor than in December 2021—the average wait time for all methods combined decreased from 2 min and 52 seconds to 44 seconds (12). From implementation to December 2022, the Lifeline has received over 2.1 million contacts, representing more than 1.43 million calls, 416,000 chats, and 281,000 texts (12).

Recent efforts have aimed to encourage more action from states, as data suggests that state investments in crisis services may impact 988 performance (for example, in-state answer rates vary widely across states from 51% to 98%, which may imply state investments are linked to crisis service performance) (12). One such effort was the Consolidated Appropriations Act (32) passed in December 2022, which included several provisions to improve coordination, standardization, and evaluation of 988 across states. Specifically, the Consolidated Appropriations Act established SAMHSA's Behavioral Health Crisis Coordinating Office and spearheaded the identification and publication of the behavioral health crisis response continuum best practices. However, given that the Consolidated Appropriations Act is still in its very early stages, it is challenging to discern how much it will move the needle. Especially in some states without long-term funding strategies, it is unclear whether, even in the short term, local Lifeline call centers can maintain their pace as federal funding decreases and demand increases (33).

### Accessibility & affordability of mental health services in Georgia

Georgia had a scopious network of call centers before the FCC designed 988 as the National Suicide Hotline number in 2020. In fact, its crisis system had been decades in the making. Since the mid-1990s, the Georgia Department of Behavioral Health and Developmental Disabilities (DBHDD) has been building a system of community-based crisis stabilization sites, including behavioral health crisis centers and crisis stabilization units, to support individuals needing psychiatric stabilization or substance use detoxification (34). In 2006, the Georgia Department of Human Resources Division of Mental Health, Developmental Disabilities, and Addictive Diseases developed a statewide suicide hotline number—the Georgia Crisis and Assess Line (GCAL) (35). The hotline was created in response to Georgia taking in evacuees from Hurricane Katrina in 2005 and attempting to help people new to the city navigate the crisis system (36). Designed to create a consistent telephonic response to those experiencing a mental health or substance use crisis, GCAL was equipped to provide assessment, brief telephonic crisis intervention, and referral services for individuals in Georgia.

The modern scaffolding for Georgia's behavioral health care system arose from a 2010 settlement agreement with the U.S. Department of Justice (DOJ). In 2009, the DOJ sued Georgia for violating the Americans with Disabilities Act (ADA) and the 1999 Supreme Court decision in *Olmstead v. L.C* (37). The plaintiffs in Georgia alleged that Tommy Olmsted, the commissioner of the Georgia Department of Human Resources, was responsible for the state's failure to serve individuals with mental illnesses and intellectual and developmental disabilities. They advocated for the right to community-based mental health care rather than institutions and hospitals. The Supreme Court held that retaining people with mental health challenges in institutional settings equated to unjustified segregation of people with disabilities, and thus a violation of Title II of the ADA (37). Georgia settled with the U.S., acknowledging its failure to serve its constituents due to the state's lack of mental health infrastructure.

The settlement agreement spelled out specific requirements for Georgia, within precise timeframes, to prevent unnecessary hospitalizations and allow people to be served in their communities (38). For example, it required Georgia to “have 18 crisis apartments,” “establish 12 crisis respite homes,” “provide peer support services to individuals,” and “establish six Crisis Services Centers by July 1, 2015,” along with a range of other crisis management services (38). In response, Georgia not only updated GCAL to provide real-time access to available crisis and detox beds throughout Georgia, but also added the MyGCAL app for text, chat, and linkage with the national Lifeline (34).

While Georgia made significant strides in behavioral health crisis services before the 988 Act passed in 2020, its services are inherently limited because it has not expanded Medicaid under the ACA (39). As of November 2023, Georgia is one of the 10 remaining states that have refused the federal government's offer to increase access to health insurance for low-income residents in exchange for federal funding (40). Georgia Governor Brian Kemp reasoned that expansion “would shift a significant number of Georgians away from private coverage, only exacerbating the financial challenges faced by hospitals” (41). Conversely, Medicaid has been shown to improve hospital finances by extending coverage to uninsured patients who would otherwise qualify for hospital charity care or be unable to pay their bills (42). Beyond reducing financial hardship, studies suggest that hospitals in Medicaid expansion states have a larger amount of mental health treatment facilities and reimbursable psychotropic medications than non-expansion states (43).

The drawbacks of non-expansion are palpable. Across Georgia, caregivers point to limited mental health resources within their communities and challenges that prevent them from efficiently securing mental health treatment, including high costs, poor coordination between providers, difficulty identifying providers that accept their insurance, and other coverage barriers (44). More than 96% (44) of Georgia's counties are designated as Mental Health Professional Shortage Areas (MHPSAs) (45). In 2019, Georgia only had one mental health care provider for every 690 people living in the state (46). The pandemic has only aggrandized pressure on what was already a strained system (44). Accordingly, the impact of GCAL can only go so far when Georgia's continuum of care is interrupted by the paucity of services.

When the 2020 Act was passed, Georgia determined that GCAL would answer calls from 988 for numbers with a Georgia area code but planned to continue promoting GCAL as the predominant form of crisis response. Mental health policymakers in the state predicted that Georgia would not see a large increase in callers because it already had a statewide system (36). Recent projections of growth in call volume to GCAL, however, tell a different story, which we will investigate further in the pre-post policy analysis.

Recently, Georgia acknowledged its need to advance behavioral health even beyond instituting a crisis system like 988. During the 2022 state legislative session, the Georgia General Assembly voted unanimously to pass the Mental Health Parity Act, which aims to hold the state accountable for enforcing parity in insurance coverage for behavioral health care for the first time (47). The Mental Health Parity Act intends to help Georgians access affordable mental health and substance use disorder treatment, with the hope that adequate reimbursement of providers by insurers will address the gaps in the mental health workforce. During the same year, the state legislature passed a bill that provides for co-responder teams composed of peace officers and behavioral health professionals. While these laws are nascent and implementation is in the early stages, they will hopefully bolster the state's future 988 crisis response.

## Pre-post policy analysis of the Lifeline's effectiveness in Georgia

The pre-post policy analysis supports this paper's research objectives by answering the following question: has the performance of the suicide and mental health crisis system in Georgia improved since the implementation of 988, and what is the law's impact in improving or not improving performance? Below, we discuss the answer to this question using publicly available data from Vibrant, SAMHSA, and DBHDD. Over the past 2 years, DBHDD has published several webinars discussing the state's rollout of 988, including preliminary data that serve as key data points in our analysis. Our analysis is stratified by the time immediately before the passage of the 2020 Act, between the Act's passage and implementation, and the months after implementation.

### Pre-passage of the 2020 Act (October 17, 2020)

In the year before the passage of the National Suicide Hotline Designation Act of 2020, Georgia Governor Nathan Deal and the state legislature included an additional $20.6 million in the proposed 2019 budget to improve and expand children's behavioral health services (48). Out of that appropriation, $1,092,000 was directed to suicide prevention efforts, which went in part toward expanding GCAL (48). Compared to the FY22 state budget, which allotted $114,000 for 988 planning, $302,000 for technology upgrades, and a combined $5 million in federal funding from the American Rescue Plan Act and SAMHSA, Georgia's funding for suicide prevention efforts in FY19 was limited (49).

Data is also limited in assessing the number of calls, texts, and chats to GCAL immediately preceding the 2020 Act. Table 1 below reflects the number of calls, texts, and chats received by GCAL call centers in Georgia in FY20 and FY21 where FY20 is based on the calculated increase in FY21 callers from DBHDD. The numbers reflect calls received by GCAL via help-seekers contacting both GCAL and 988, as calls to 988 were (and still are) routed through GCAL (50).

**Table 1:** Comparison of callers into Georgia's lifeline number, FY20 and FY21.

	**Pre-passage (FY20)**	**Post-passage/ Pre-implementation (FY21)**
Calls, texts, and chats received by GCAL + 988 (49)	209,000 calls, texts, and chats (49)	275,000 calls, texts, and chats (49)

In a perfect world, the 24% increase in number of calls, texts, and chats in FY21 compared to FY20 would prove the passage of the National Suicide Hotline Designation Act of 2020 alone was effective in expanding the number of people accessing suicide prevention services. To make this assertion, historical growth trends or growth trends that would have been anticipated even in the absence of the transition to 988—must be assessed. According to SAMHSA, from calendar year 2007 to 2020, national call volume to the Lifeline increased an average of 14% per year, reflecting the ongoing promotion of the Lifeline by mental health and suicide prevention organizations (3). The number of total calls, texts, and chats to GCAL and 988 in FY21-−275,000 in total or a 24% increase from FY20—represents the highest call volume since GCAL's inception (49). While historical trends for GCAL were not accessible, the surge in call volume in Georgia after the passage of the Act suggests a higher growth rate than the 14% calculated by SAMHSA.

One explanation for the increase in call volume could be that it reflects the increase in the national suicide rate at the time. If this were the case, the swell in callers would track alongside suicidality rates nationally. However, the national suicide rate increased by 4% between 2020 and 2021 (51), while the increase in callers to GCAL was six times this percentage (noting that FY20 and calendar year 2020 and FY21 and calendar year 2021 is not exact, but sufficient for the purposes of this paper). This points to more people potentially calling the Lifeline because of the FCC's passage of the 988 legislation and the political pressure put on states to ramp up the delivery of suicide prevention services.

### Pre-implementation of the 2020 Act (October 17, 2020–July 16, 2022)

After the Act was passed on October 17, 2020, Georgia had 20 months to prepare for the rollout of 988. The 2020 Act required Georgia to enhance its current system's ability to respond to those in crisis by ensuring a call-taker was available 24/7 to respond to calls, texts, and chats; offering peer-run warm lines for emotional support; establishing additional mobile crisis units, crisis service centers, crisis stabilization units, inpatient beds, outpatient services, and detox and substance abuse disorder treatment statewide; and coordinating with 911 and emergency medical services when appropriate (49).

Instead of implementing monthly fees on telecommunications bills to pay for 988-related expenditures, Georgia leveraged one-time COVID-19 relief funds and available state dollars to prepare for the launch (52). Specifically, Georgia invested $20.5 million—a combination of appropriations from the General Assembly and federal COVID-relief funds in 988 related expenditures (52). Further, SAMHSA announced in December 2021 that Georgia would spend approximately $3,756,750 on 988 Lifeline implementation with $996,008 for other crisis-related services over the next 4 years in Mental Health Services Block Grants (MHBG), MHBG-COVID funds, and MHBG-American Result Plan (ARP) funds (3).

**Figure 1:**
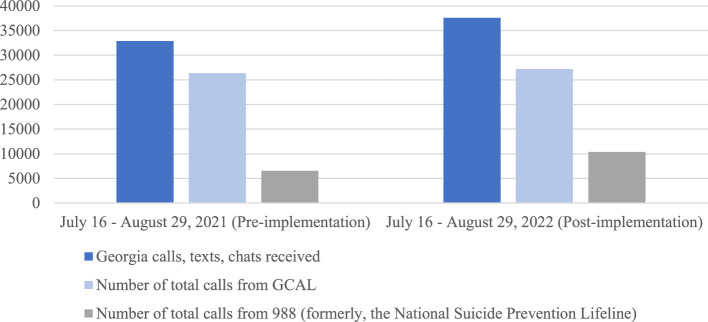
Comparison of calls, texts, and chats in the first 45 days of 988 rollout (16).

Figure 1 shows that the majority of outreach to the Lifeline before implementation between July 16 and August 29, 2021, were received by GCAL (80.19%) (16). This decreased immediately after implementation (72.39%) during the same time period in 2022 (16). Total calls, texts, and chats rose from 32,843 between July 16 and August 20, 2021, to 37,561 in 2022, representing a 14% increase (16). The upsurge in callers may indicate that post-passage and pre-implementation, national efforts to educate people about the Lifeline were underway, as federal and state dollars were already flowing into establishing the number and advertising it as a resource. It may also reflect that the easy-to-remember 988 number helped socialize information about suicide prevention resources. Other explanations for the increase in call volume to GCAL could be a direct result of the impact of COVID-19, the rise in opioid-related drug overdoses, and other sociocultural factors such as living in rural vs. urban areas, occupational hazards, sexual identity, social media, and more.

### Post-implementation of 2020 Act (July 16, 2022)

The 2020 Act was nationally implemented on July 16, 2022 nearly 2 years after its passage. Implementation refers to the deadline indicated in the Act by which states must implement the infrastructure to provide 988 services. The data in Table 2 illustrates a direct comparison between calls, texts, and chats received to GCAL in October through December 2021, before 988 implementation, compared to the same time 1 year later, after the official 988 implementation as designated in the Act. Table 2 also includes information related to the average in-state 988 answer rate and the average in-state call abandon rate using data consolidated by Vibrant. The abandon rate means the number of calls that disconnect prior to being engaged by a counselor at a state or territory's centers. Disconnection may happen for several reasons if the caller changes their mind about seeking care at that moment, no longer feels they have privacy or safety in their environment, or experiences a technical service interruption, which may occur due to internet instability or carrier glitches (54).

**Table 2:** 988 outreach volumes and answer rates, October–December 2021 vs. 2022.

	**October–December 2021 (pre-implementation)**	**October–December 2022 (post-implementation)**
Calls, texts, and chats received by a GCAL call center (53)	63,314	69,380
Average in-state 988 answer rate (“Answered In-State” calls divided by all calls “Received” to the state) (54)	63%	81.33%
Average in-state abandoned calls (54)	1,241.33	624.33

On first glance, the performance of 988 in Georgia between October and December 2021 to October through December 2022 is encouraging. The number of calls grew from 63,314 to 69,380, representing a nearly 10% increase in total number of callers. Table 2 also indicates a surge in the average in-state 988 answer rate, representing a nearly 30% increase. The reduction in abandoned calls tracks alongside the answer rate after 988 implementation, with a sharp decrease in the number of received calls that disconnect prior to engaging with a trained call-taker. While there are a variety of reasons for disconnect, including the caller changing their mind about receiving care, technical glitches in the system, service interruption, and more, the lower number of abandoned calls likely points to more people receiving care from a trained call-taker.

While the numbers in Tables 1, 2, Figure 1 paint a picture of how the 2020 Act impacted Georgia's expansion of 988 based on available information, they do not include the multiple confounding factors that may affect volume, such as the extent and duration of public promotion and marketing of 988. Significant increases in call volume have also been observed with major media attention devoted to the Lifeline number. For example, in 2017, American hip-hop artist Logic released a song called “1-800-273-8255,” named after the 10-digit Lifeline number. One study suggests that Logic's song was responsible for a 6.9% increase in calls to the Lifeline during the 34-day period when public attention to the song was substantial (55). Although the 2020 Act made way for additional funding to expand access to crisis services, in many ways, incorporating 988 into the 10-digit Lifeline number landscape was little more than an advertising scheme. An easy-to-remember three-digit number is far simpler to communicate than the previous 10-digit Lifeline number. Though it is unclear at this point the extent in Georgia (given the state's 988 marketing campaign, to date, is not yet underway), broadly advertising the Lifeline has influenced help-seeking behaviors among callers.

The political dynamics around 988 implementation add further nuance to this story. Policymakers have admitted there was a period where GCAL was not picking up calls from the national Lifeline. When 988 was first rolled out, centers were getting paid to answer the local line, but not the national Lifeline. In fact, some would get penalized if they answered national calls, so they would prioritize local calls. It was not until funding arrived for 988 that call centers, like GCAL, had more agency to answer 988 calls. Thus, the increase in the percentage of calls into GCAL from 988 may not be explained by the increase in call center capacity as a direct result of the new legislation, but rather by people calling 988 no longer being deprioritized and neglected in state call centers. This adds texture to our understanding of the interplay of federal vs. state governments in improving 988 performance.

Nonetheless, the pre-post analysis points to a correlation between the performance of the Lifeline in Georgia and the implementation of 988, given the surge in callers (the increased number of people who know about the Lifeline and use its services) and the increased answer rate (representing more people receiving mental health crisis services from qualified call-takers) after implementation. Still, it is critical to note the major investment needed to ensure uninterrupted continuity of care. Although previous studies suggest that 80% of calls can be resolved without dispatching mobile crisis teams, the remaining 20% who need crisis services will only get larger with higher volumes of callers (3). Federal projections for Georgia estimate that 56,460 mobile crisis responses were dispatched in FY23, which is an increase of 176% from FY21 (49). Accordingly, bed capacity in Georgia would have needed to increase by 105% to address the 67,137 predicted admissions to community crisis beds in FY23 (up from 32,700 in FY21) (49).

While funding has expanded between 2022 and 2023 (49), sustainable financing mechanisms must be implemented in Georgia to account for the increased capacity. We suggest several policy recommendations in the section below to ensure that well-trained personnel can provide future callers with adequate, affordable services from the moment they call 988 to when they are released from a crisis center, should their mental health require it.

## Results of pre-post analysis: policy recommendations

Based on the results of the pre-post analysis, we have identified Georgia-specific policy recommendations to improve 988 performance and ensure more people can access the mental health care they need. The three key recommendations for Georgia include: (1) address the statewide mental health workforce scarcity, (2) sustainably increase funding for 988, and (3) consider expanding Medicaid to ensure more people at and below the poverty line can equitably access services. In addition, we underscore several key learnings from Georgia that are critical to the future success of 988 nationwide.

First, Georgia must address its current behavioral health workforce scarcity, especially given the increase in number of potential care-seekers because of 988 (56). The pre-post policy analysis makes clear that the number of people contacting GCAL and 988 via call, text, and messaging will increase, especially after Georgia begins its 988 marketing campaign in late-2023 (49). As of 2022, 76 of Georgia's 159 counties did not have a licensed psychologist and 52 counties were without a licensed social worker (57). These numbers are lower than the national average. According to the 2022 Report published by the Georgia Mental Health Policy Partnership and Behavioral Health Services Coalition, Georgia ranked 48^th^ among all states in access to mental health care (57). Without increasing the number of mental health professionals in the state proportionally, Georgia will see wider gap between the number of care-seekers and the number of professionals able to provide services. Demand, as a result, will only continue to exceed capacity.

Georgia not only must find ways to increase its behavioral health professional capacity, but also the state has a duty to increase call center capacity both for Georgia constituents and callers nationwide. If the state call center does not have the capacity to support a caller, that caller will be transferred to a national call center. This may result in long wait times when seconds and minutes—let alone hours may mean the difference between life and death. Even more, when a person, regardless of their area code, texts, or chats 988, the message will be directed to whichever state is next in the queue to respond via the federally funded call center (3). This is important because if the number of people texting or chatting from Georgia spikes dramatically without corresponding increases in state message-taking capacity, it prevents people nationwide from getting the same level of service as those calling in. While this system will ideally improve with more funding, in the interim, it represents a patchwork solution and furthers the argument of ramping up capacity.

Thus, to expand the behavioral health workforce and call center capacity, there are several measures Georgia and states generally can take. Legislatures should make it more economically feasible for people to become mental health providers and stay in their roles despite the exhaustion that results from an extreme lack of capacity. For example, in Georgia, policymakers can provide loan forgiveness for those who work in areas impacted by workforce shortages, explore opportunities to develop and implement state loan repayment programs (such as Physicians and Dentists Rural Assistance Programs), and expedite the licensure of mental health clinicians, including qualified foreign-born clinicians who can help develop a culturally competent behavioral health workforce (57). Evidence-based, cost-effective interventions can expand the behavioral health workforce and empower the community. For instance, community-initiated care is a concept that depends on the “task-shifting” model of services. In this way, mental health care is not dependent on licensed clinicians but rather on non-specialized healthcare workers and even lay members of the community who are trained with the knowledge, skills, and competencies necessary to deliver behavioral health support (58).

Georgia must also consider ramping up the number of call-takers within GCAL and 988 call centers. While projecting changes in the volume of callers into 988 presents difficulties and therefore makes it challenging to predict exact staffing needs, the trends thus far indicate that expanding capacity is critical. Call centers in Georgia and nationwide must be mindful of recruitment, training, and retention among call-takers. Strategies to increase workforce capacity include accelerating the license review process for behavioral health providers, allocating more funds to salary raises, and providing flexible and remote work options for call-takers. By investing in behavioral health and call center infrastructure, states can enable more people in crisis who call 988 to receive mental health care—reducing suicide rates overall and promoting a more mentally healthy society.

Next, policymakers cannot discuss increasing capacity in Georgia without a discussion of funding. Given that money for Georgia's 988 rollout currently comes from COVID-19 emergency relief efforts, the state needs a sustainable financing mechanism to continue paying for the Lifeline after emergency funds dry up. Multiple sources of funding could be used to support the anticipated demand for Lifeline services. On the federal side, the SAMHSA Suicide Lifeline grant supports the infrastructure of network operations, and the MHBG funds provide technical assistance on the use of funds, allocations of funding, and recommended changes to the data reporting system. Georgia can also impose and collect telecommunication fees to support 988 operations authorized by the 2020 Act. The fees would be collected from each subscriber of commercial landline telephones, cellphones, and IP-enabled voice services, and the revenue generated would be expanded only in support of 988 services or enhancements of such services. In a recent presentation by DBHDD, one representative stated, “While other states have rushed to pass fees, Georgia leaders will continue to assess actual call volume to determine how best to approach funding this long-term transformation of Georgia's crisis infrastructure” (49). However, early adopters of telecom fees, such as Virginia and Washington, reported collecting between 3.6 and 4.5 million in 988 telecommunication fees during FY2021 (12), suggesting that passing legislation enabling fees generates significant revenue to fund the call line (59). Thus, without state legislation and adequate funding and staffing of the Lifeline, Georgia—along with the majority of other states that have not passed telecom fees—cannot convalesce its crisis response performance and continuum of care.

Some states have used Medicaid to support elements of the crisis continuum and expand capacity through plan amendments, waivers, and demonstrations. Medicaid managed care payers cover several aspects of crisis services—more typically crisis intervention and stabilization services, not call response. To date, private payers have provided limited coverage of crisis services (3). However, as a non-Medicaid expansion state, Georgia's ability to explore the use of Medicaid funding is limited.

As such, our final recommendation requires Georgia and other non-Medicaid expansion states to assess their Medicaid status. Ramping up Georgia's behavioral health crisis system requires not only increasing capacity, such as allocating additional funding to expand the mental health professional workforce, but also it means ensuring that people across the state can receive adequate and affordable care. Studies show that without sufficient participation in Medicaid among psychiatrists, Medicaid enrollees with behavioral health needs may be unable to find a doctor who accepts Medicaid patients and, even if they do, likely experience long waits for intake appointments (60).

Expanding Medicaid in Georgia would mean that the income threshold for Medicaid would increase, allowing more people who make at or below the poverty line to receive coverage. By enabling Medicaid to cover a higher percentage of people, Georgia can ensure that more of its constituents have access to affordable behavioral health services. The four other states that, like Georgia, have uninsured rates of 12% or more—Florida, Oklahoma, Texas, and Wyoming have not expanded Medicaid eligibility and may benefit from following suit (61).

Even more, evidence shows that Medicaid coverage expansion improves access to care and medications for those with behavioral health challenges (62). When more people have access to affordable and quality care, they also have access to preventative health screenings, leading to a decrease in delaying and forgoing necessary care altogether. Coverage expansion contributes to widened access to behavioral health services by increasing mental health provider capacity. The more likely providers are to receive adequate reimbursement for their services, the greater their capacity to accept various forms of coverage. With greater access to affordable mental health services, people are less likely to delay seeking treatment until they are in crisis.

Georgia recently narrowly expanded Medicaid to cover more low-income adults in its “Pathways to Coverage” program but limited it to individuals working or volunteering for at least 80 h per month. Experts at George Washington University argue that this legislation both costs more and covers fewer people than a full Medicaid expansion, as it resulted in the denial or termination of anyone failing to document 80 h of work or equivalent activities *every* month (63). As a result, it excludes hundreds of thousands of eligible Georgians from the assistance they would otherwise receive under the ACA and creates a narrow coverage pathway only few can navigate. Thus, Georgia must consider further legislation to close the Medicaid coverage gap to improve access to needed mental health care.

## Discussion

Overall, this analysis shows that the performance of the crisis system in Georgia improved since the implementation of 988. That there was an existing behavioral crisis mechanism in place given the already-developed GCAL network likely allowed Georgia to address increased call volumes more quickly. However, sustainable state funding mechanisms must be instituted to ensure the sustainability and effectiveness of the Lifeline going forward. Further, Georgia's non-Medicaid expansion state designation prevents a coordinated continuum of care, as many people do not have access to affordable behavioral health services and refrain from engaging with the broader healthcare system as a result. Thus, Georgia along with many other states in similar positions must formalize additional funding sources and ensure there is an adequate workforce to address growing behavioral health needs.

### Limitations of the pre-post policy analysis

There are unavoidable limitations in our pre-post analysis and policy recommendations. First, and most notable, is the lack of data. Several policy experts with whom we consulted in drafting this paper made clear that the lack of publicly available data is deeply troubling. While call volume, wait times, and other metrics from 988 provide some insights into accessibility and demand for 988, they do not tell the whole story. State-to-state comparisons could be useful for understanding the difference between successful strategies and interventions that fall short. For example, a deep dive into Medicaid expansion states' crisis response and continuum of care could provide useful metrics for non-Medicaid expansion states, like Georgia. Furthermore, specific research evaluating the equitable impact of 988 on vulnerable and minority populations, especially populations living with disabilities and Veteran populations, could benefit communities that often have the most challenging experiences accessing services. Additional state and national crisis center metrics help inform the 988 implementation and future program improvements and allow researchers to understand how 988 has impacted the continuity of care.

Moreover, geolocation obfuscates the 988 policy recommendations. When a user calls 988, they are routed to a local call center based on their area code vs. their geographic location (33). Area code routing results in less precise emergency response service deployment and coordination, and call center operators tend to be less knowledgeable about care systems outside their state (33). According to mental health and crisis counseling experts, getting the caller to the geographically appropriate local crisis center is key to the Lifeline's approach to providing services to those in need of public health and safety resources (64). However, many policymakers and advocates oppose geolocation as a requirement for 988 implementation, citing issues related to privacy and confidentiality (65).

Consequently, the numbers used in the pre-post policy analysis do not demonstrate if callers into Georgia's lifeline require crisis services in the state of Georgia or elsewhere. If most people calling with Georgia area codes are physically in locales outside of Georgia, the policy recommendations would likely look different. While Georgia has considered leveraging currently available 911 capabilities and infrastructure to serve as a model for Lifeline geolocation, most states have not (49).

Further, this analysis does not stratify by different demographic groups who may receive specialized services via 988. For example, Veteran or active military callers can dial 988 then press 1, which connects them to the Veteran Crisis Line, and American Indian and Alaska Native Communities can dial 988 then press 4 to be connected to the Native and Strong Lifeline. Thus, future research can look to other crisis lines such as the National Domestic Violence Hotline and offshoots of 988 such as the Veteran Crisis Line and the Native and Strong Lifeline to understand the hurdles implementers and policymakers have considered in addressing specific populations (66).

Relatedly, there are several outcomes this paper could have evaluated but did not. Such outcomes include assessing the impact of 988 on mental health stigma, health-seeking behavior, young adult mental health, and more. Provided that the laws are nascent, there will likely be other outcomes to evaluate that are not yet on researchers' and policymakers' radars. Future research papers could address such outcomes.

### National implications

Has the performance of the suicide and mental health crisis system in the U.S. improved since the implementation of 988? For the reasons stated above, Georgia offered us insight into the national implications of the 988 crisis line given its designation as a nationally representative state, providing a window into how states can assess progress and shortcomings of the Lifeline. Still, the impact and effectiveness of 988 on a national level remains one of the largest open questions in crisis intervention services in the U.S. (67). While researchers have begun looking into the effectiveness of third-party callers (68) and chat interventions (69) and preliminary implementation guidance has been published by SAMHSA (3), peer-reviewed literature examining the nationwide effectiveness of the 988 suicide hotline post-rollout has yet to be published.

Nonetheless, there have been studies that evaluated the success of 988 on a national scale before the number was implemented. For example, a recent national survey of 180 behavioral health directors assessed whether they felt prepared for a national transition to 988 in the lead-up to implementation (70). Survey respondents stated that the most common challenges they encountered in their 988-planning process were insufficient mental health workforce and a lack of funding (70), both of which were found in our analysis of Georgia as well. The researchers found that local, state, and regional behavioral health systems across the country require greater investments to support 988 and mental health crisis care, regardless of whether they have expanded Medicaid under the ACA (70). However, the states that have not expanded Medicaid under the ACA, like Georgia, create additional barriers for people to access affordable and quality mental health care.

Beyond evaluating 988 for its effectiveness in preventing suicide, researchers may also look to existing national hotlines targeting issues such as child abuse, child trafficking, and domestic violence, which, similar to mental health crises, tend to be socially condemned and stigmatized. Evaluation of such national hotlines may offer researchers a glimpse into effective strategies to improve hotline performance on a national level for socially treated issues. Additionally, given that suicide is higher among violence-involved individuals (71), hotlines like the National Domestic Violence Hotline and the Intimate Partner Violence hotline may attract similar callers. Training national hotline workers who support violence-involved individuals in suicide prevention strategies and referral to care may also be an effective way to approach mental health crises and should be explored further (71).

### Call to action

The interplay between the federal and state governments in Georgia largely works to improve the performance of the Lifeline. Still, with the federal government's plan to market 988 more broadly in 2023, more states will require the government's guidance on how to advertise 988 on a local level. With a more sophisticated system often comes more complexity. National, state, and local governments must ensure that each is not operating in a silo and work in tandem with stakeholders, such as law enforcement. More research that proves the Act's effectiveness will hopefully provide a positive reinforcement loop to encourage continued legislative action.

It is vital to note that while the 2020 Act was a step in the right direction in enforcing mental health parity, it is by no means enough. Federal, state, and local governments have a necessary role to play in providing affordable and accessible mental health care to their constituents. However, governments should not focus solely on addressing moments of crisis. Ultimately, in addition to 988, governments must also invest in in pre-crisis mental health care. Public education campaigns should inform people about warning signs to help identify mental health challenges in the early stages. Schools should implement mental health and drug use screening protocols and require the presence of emotional support systems for students. Availability of warm lines should be expanded to prevent full-blown crises and offer post-crisis counseling and care. And, of course, this should all be done while ensuring people with lived experience have a seat at the decision-making table.

The nation's focus on suicide prevention is a key step forward. But, waiting until suicidal ideation or attempts occur to act is indicative of a system that has already failed its constituents. 988 will be a letdown—not a lifeline—if policymakers wait for suicide to act.

## Author contributions

MB: Conceptualization, Data curation, Formal analysis, Investigation, Methodology, Writing – original draft, Writing – review & editing. JS: Conceptualization, Methodology, Supervision, Writing – review & editing.
